# Machine‐Reported Electrocardiographic Right Axis Deviation or Right Ventricular Hypertrophy and Echocardiographic Pulmonary Hypertension Phenotypes and Right‐Heart Abnormalities

**DOI:** 10.1111/echo.70539

**Published:** 2026-07-13

**Authors:** Yoshihiro Fuchigami

**Affiliations:** ^1^ Faculty of Medicine Kyushu University Fukuoka Japan

**Keywords:** echocardiography, electrocardiography, pulmonary hypertension, right axis deviation, right ventricular hypertrophy, tricuspid regurgitation velocity

## Abstract

**Purpose:**

Machine‐reported electrocardiographic findings suggesting right‐heart strain are routinely generated during electrocardiogram (ECG) acquisition, but their relationship to echocardiographic pulmonary hypertension phenotypes and right‐heart abnormalities remains incompletely defined. This study evaluated whether machine‐reported right axis deviation (RAD) or right ventricular hypertrophy (RVH) is associated with echocardiographic pulmonary hypertension phenotypes and right‐heart abnormalities in hospitalized patients.

**Methods:**

This retrospective observational study used linked MIMIC‐IV‐ECG and MIMIC‐IV‐ECHO data. Echocardiograms were linked to the closest ECG obtained from 7 days before echocardiography through the time of echocardiography. The primary exposure was machine‐reported RAD or RVH. The primary outcome was Echo‐PH specific, a composite echo‐derived phenotype including tricuspid regurgitation velocity greater than 3.4 m/s, moderate or severe pulmonary hypertension text, or right ventricular pressure overload. Logistic regression adjusted for demographics, comorbidities, and ICU status, with patient‐level cluster‐robust standard errors.

**Results:**

The cohort included 68 905 ECG–echocardiography pairs from 42 078 patients; 2815 pairs were RAD/RVH‐positive. For Echo‐PH specific, RAD/RVH had sensitivity 7.5%, specificity 96.7%, positive predictive value 35.5%, and negative predictive value 81.4%. RAD/RVH remained associated with Echo‐PH specific after full adjustment using cluster‐robust standard errors (odds ratio, 2.04; 95% confidence interval, 1.84–2.26). The adjusted probability of Echo‐PH specific was 18.8% without RAD/RVH and 30.0% with RAD/RVH.

**Conclusion:**

Machine‐reported ECG RAD/RVH was highly specific but insensitive for echocardiographic pulmonary hypertension phenotypes and right‐heart abnormalities. These routine ECG findings may serve as supportive clues to echocardiographic right‐heart disease but should not be used to exclude it.

## Graphical abstract

Machine‐reported ECG right axis deviation or right ventricular hypertrophy was highly specific but insensitive for echocardiographic pulmonary hypertension phenotypes and right‐heart abnormalities. These routine ECG findings may serve as supportive clues, but should not be used to exclude echocardiographic right‐heart disease.

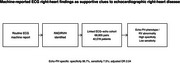

## 1 Introduction

Pulmonary hypertension and right‐heart abnormalities are clinically important findings associated with impaired right ventricular adaptation and adverse outcomes. Although pulmonary hypertension is defined hemodynamically by right heart catheterization, transthoracic echocardiography is widely used as a noninvasive tool to estimate pulmonary pressures and evaluate right‐heart structure and function [5, 6]. Current guidelines emphasize tricuspid regurgitation velocity and comprehensive right‐heart assessment, including right ventricular size and function, right atrial size, inferior vena cava findings, and tricuspid regurgitation severity [5, 7, 8, 10, 11]. Large echocardiographic cohorts have shown that even mild elevations in estimated pulmonary pressure are associated with mortality and right ventricular dysfunction [12, 13]. Right ventricular adaptation and dysfunction are central determinants of prognosis in pulmonary hypertension [14, 15, 16].

Electrocardiographic findings such as right axis deviation (RAD), right ventricular hypertrophy (RVH), right atrial enlargement, and right bundle branch block have long been considered potential markers of right‐heart strain. In pulmonary hypertension cohorts, electrocardiogram (ECG) findings such as RAD and RVH‐related criteria have generally shown high specificity but limited sensitivity for pulmonary hypertension or severe pulmonary hypertension [17, 18, 19, 25]. In patients with pulmonary hypertension or right ventricular remodeling, studies using cardiac magnetic resonance imaging have similarly shown that ECG criteria have limited sensitivity for RVH or dilation [20, 21, 22, 23]. These findings suggest that ECG abnormalities may support suspicion of right‐heart disease when present, but their absence does not reliably exclude pulmonary hypertension or right ventricular abnormality.

Recent waveform‐based ECG prediction studies have demonstrated that ECG waveforms contain diagnostic information related to pulmonary hypertension and pulmonary vascular disease [26, 27, 28, 29, 30]. These approaches have shown promising discrimination for pulmonary hypertension detection and risk assessment, particularly when ECG data are combined with other clinical or imaging modalities. However, dedicated prediction models are not necessarily available in routine clinical workflows. In contrast, machine‐generated ECG interpretation text is already widely produced during standard ECG acquisition, yet the clinical meaning of routine machine‐reported right‐heart findings in relation to echocardiographic pulmonary hypertension phenotypes and right‐heart abnormalities remains insufficiently characterized.

The availability of linked MIMIC‐IV‐ECG and MIMIC‐IV‐ECHO datasets provides an opportunity to evaluate this question in a large real‐world cohort. MIMIC‐IV‐ECG includes diagnostic 12‐lead ECGs with machine‐generated report text linked to clinical data, while MIMIC‐IV‐ECHO provides structured echocardiographic measurements and findings [1, 2, 3, 4]. Therefore, this study investigated whether machine‐reported RAD or RVH on routine ECG was associated with echocardiographic pulmonary hypertension phenotypes and right‐heart abnormalities in hospitalized patients. The primary hypothesis was that these ECG findings would be highly specific but insensitive supportive clues for echocardiographic right‐heart disease.

## 2 Methods

### 2.1 Study Design and Data Sources

This retrospective observational study used linked data from MIMIC‐IV, MIMIC‐IV‐ECG, and MIMIC‐IV‐ECHO. MIMIC‐IV is a publicly available, deidentified electronic health record database derived from patients treated at Beth Israel Deaconess Medical Center, with data organized into hospital and intensive care modules [2]. MIMIC‐IV‐ECG contains diagnostic 12‐lead electrocardiograms linked to MIMIC‐IV patients and includes machine‐generated ECG report text in the machine measurements table [3]. MIMIC‐IV‐ECHO contains structured echocardiographic measurements and findings linked to the MIMIC‐IV clinical database [4]. Data were accessed through PhysioNet after completion of the required credentialing process and data use agreement [1].

### 2.2 Study Population

The source population consisted of patients with at least one structured echocardiographic study in MIMIC‐IV‐ECHO and at least one diagnostic 12‐lead ECG in MIMIC‐IV‐ECG. For the primary analysis, each echocardiographic study was linked to the closest ECG performed from 7 days before echocardiography through the time of echocardiography. ECGs obtained after the echocardiographic study were not used in the primary analysis to preserve the temporal ordering of ECG findings before echocardiographic assessment. When multiple ECGs met the matching criterion for the same echocardiographic study, the ECG closest in time to the echocardiogram was selected.

**FIGURE 1:**
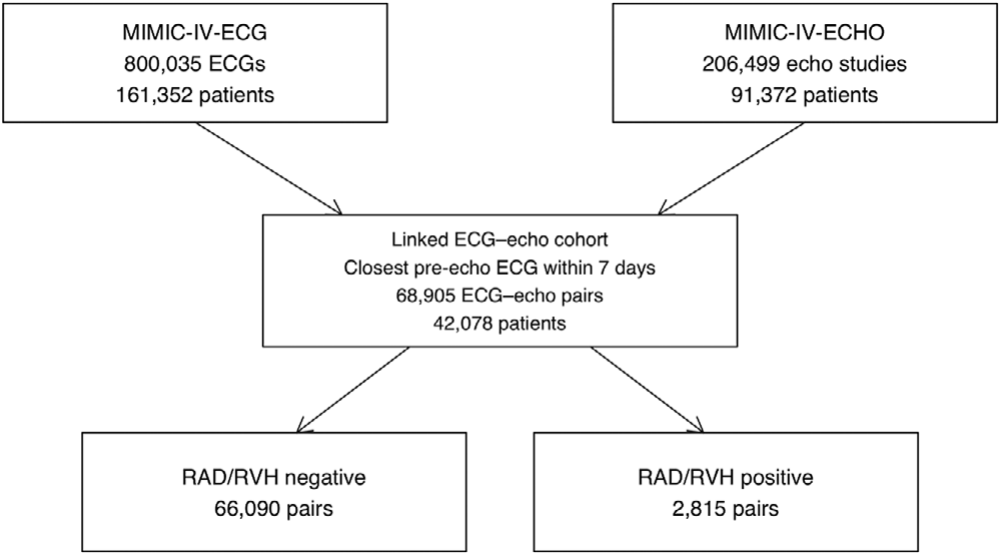
Study flow diagram. Flow diagram showing derivation of the primary electrocardiogram (ECG)–echocardiography cohort from linked MIMIC‐IV‐ECG and MIMIC‐IV‐ECHO data. The primary analysis included the closest ECG obtained from 7 days before echocardiography through the time of echocardiography.

The final primary analytic cohort included 68 905 ECG–echocardiography pairs from 42 078 patients. Of these, 2815 pairs were positive for the primary ECG exposure and 66 090 were negative. The derivation of the cohort is shown in Figure 1.

### 2.3 ECG Exposure Definition

The primary exposure was machine‐reported RAD/RVH, defined as the presence of machine‐reported RAD or RVH. Machine‐generated ECG report lines were extracted from the MIMIC‐IV‐ECG machine measurements table and concatenated across available report fields. Text was converted to lower case, and predefined terms were searched using regular expressions.

RAD was defined by explicit machine‐reported terms indicating RAD or rightward axis. RVH was defined by explicit report terms indicating RVH or RVH. RVH was defined from the machine‐generated ECG report text and did not rely on manual voltage criteria or centralized waveform interpretation. The primary ECG exposure was defined as the presence of either RAD or RVH. For clarity, this composite exposure is referred to as machine‐reported RAD/RVH.

The composite RAD/RVH exposure was specified before the final adjusted, diagnostic performance, and sensitivity analyses because RAD and RVH are established, routinely reported, and clinically interpretable ECG findings related to right‐heart strain.

Secondary ECG findings included RAD alone, RVH alone, QRS axis greater than 110°, and right bundle branch block. QRS axis greater than 110° was derived from the machine‐measured QRS axis when available.

Report fields from the same ECG were concatenated and evaluated at the ECG‐study level, so repeated mentions within the same ECG contributed only once to the ECG‐level exposure. The automated extraction was intentionally based on explicit machine‐reported terms. Because uncertain RVH wording could be captured by broad text matching, a validation review was performed using predefined adjudication rules in which borderline, possible, probable, or otherwise uncertain statements were not considered definite positive findings. Negated statements were not adjudicated as positive findings in the validation review.

### 2.4 ECG Text Extraction Validation

To assess the validity of the automated ECG text extraction, a random sample of 200 ECG report texts, including 100 RAD/RVH‐positive and 100 RAD/RVH‐negative reports according to the automated extraction, was independently reviewed using predefined adjudication rules. Definite mentions of RAD, rightward axis, RVH, or RVH were classified as positive. Borderline, possible, probable, or otherwise uncertain statements were not considered definite positive findings. Negated statements were not classified as positive findings.

### 2.5 Echocardiographic Outcomes

The primary outcome was echocardiographic pulmonary hypertension using a specific definition, hereafter referred to as Echo‐PH specific. Because right heart catheterization data were not available as the reference standard, the outcome was not defined as confirmed pulmonary hypertension. Instead, outcomes were defined as echocardiographic phenotypes consistent with elevated pulmonary pressure or right‐heart abnormality.

Echo‐PH specific was defined using a more specific composite of echocardiographic findings, including tricuspid regurgitation velocity greater than 3.4 m/s, structured report evidence of moderate or severe pulmonary hypertension, or right ventricular pressure overload. This definition was intended to identify echocardiographic findings more strongly suggestive of elevated pulmonary pressure.

Secondary outcomes were Echo‐PH primary, Echo RV abnormality, tricuspid regurgitation velocity‐only outcomes, and component right‐heart abnormalities. Echo‐PH primary was a broader echocardiographic pulmonary hypertension definition incorporating tricuspid regurgitation velocity greater than 2.8 m/s, elevated tricuspid regurgitation‐derived pressure gradient or estimated pulmonary pressure when available, structured pulmonary hypertension text, or right ventricular pressure overload. Echo RV abnormality was defined as the presence of at least one right‐heart structural or functional abnormality, including right ventricular dysfunction, right ventricular dilation, right atrial enlargement, moderate or severe tricuspid regurgitation, inferior vena cava dilation, or right ventricular pressure or volume overload.

Tricuspid regurgitation velocity‐only sensitivity outcomes were defined as tricuspid regurgitation velocity greater than 2.8 m/s and greater than 3.4 m/s among studies with available tricuspid regurgitation velocity. These thresholds were selected to evaluate whether the main findings persisted when using Doppler‐derived measurements rather than composite structured outcomes. Contemporary pulmonary hypertension guidelines emphasize tricuspid regurgitation velocity as a key component of echocardiographic pulmonary hypertension probability assessment, although echocardiography does not replace right heart catheterization for definitive diagnosis [5, 7, 11].

### 2.6 Covariates

Clinical covariates were obtained from the MIMIC‐IV hospital and intensive care modules. Demographic variables included age, sex, and race. Age was calculated using MIMIC‐IV anchor age and anchor year. Race was categorized based on admission records.

Comorbidities were identified using ICD‐9 and ICD‐10 diagnosis codes from hospital diagnoses. Prespecified comorbidities included hypertension, diabetes mellitus, heart failure, chronic obstructive pulmonary disease, chronic kidney disease, atrial fibrillation, coronary artery disease, and pulmonary embolism. ICU status at the time of echocardiography was defined by overlap between the echocardiography time and ICU stay intervals in the MIMIC‐IV ICU module.

### 2.7 Statistical Analysis

Baseline characteristics were summarized overall and according to primary ECG exposure status. Continuous variables were summarized as median with interquartile range, and categorical variables as counts with percentages. Diagnostic performance of ECG findings for echocardiographic outcomes was assessed using sensitivity, specificity, positive predictive value, negative predictive value, positive likelihood ratio, and negative likelihood ratio with 95% confidence intervals.

Associations between machine‐reported RAD/RVH and echocardiographic outcomes were estimated using logistic regression. Because multiple ECG–echocardiography pairs could be contributed by the same patient, fully adjusted models were estimated with patient‐level cluster‐robust standard errors. The fully adjusted model included age, sex, race, hypertension, diabetes mellitus, heart failure, chronic obstructive pulmonary disease, chronic kidney disease, atrial fibrillation, coronary artery disease, pulmonary embolism, and ICU status at echocardiography.

To support clinical interpretation and avoid overemphasis on odds ratios for common outcomes, adjusted marginal probabilities, risk differences, and risk ratios were estimated from the fully adjusted logistic models. Confidence intervals for these marginal estimates were obtained using patient‐level cluster bootstrap resampling with 300 resamples.

The primary analysis used the closest ECG performed from 7 days before echocardiography through the time of echocardiography. ECG–echocardiography time intervals and tricuspid regurgitation velocity availability were summarized descriptively. Sensitivity analyses evaluated alternative ECG–echocardiography matching windows, including ECG within plus or minus 1 day, plus or minus 3 days, and plus or minus 7 days of echocardiography. Patient‐level sensitivity analyses were performed by restricting the cohort to one echocardiographic study per patient using two approaches: the first echocardiogram per patient and the ECG–echocardiography pair with the shortest absolute time difference per patient. Additional sensitivity analyses were performed among studies with available tricuspid regurgitation velocity using tricuspid regurgitation velocity greater than 2.8 m/s and greater than 3.4 m/s as outcomes.

All analyses were conducted using R. A two‐sided *p* value less than 0.05 was considered statistically significant.

### 2.8 Ethics

MIMIC‐IV, MIMIC‐IV‐ECG, and MIMIC‐IV‐ECHO are deidentified datasets available through PhysioNet under credentialed access. The present study used deidentified secondary data and was conducted in accordance with the PhysioNet data use agreement. Because the data are deidentified and publicly available to credentialed users, additional institutional review board approval was not required for this secondary analysis [1, 2, 3, 4].

## 3 Results

### 3.1 Study Cohort

Among linked MIMIC‐IV‐ECG and MIMIC‐IV‐ECHO data, the primary analytic cohort included 68 905 ECG–echocardiography pairs from 42 078 patients, using the closest ECG obtained from 7 days before echocardiography through the time of echocardiography. Machine‐reported RAD/RVH was present in 2815 ECG–echocardiography pairs and absent in 66 090 pairs (Figure 1).

The median age of the cohort was 68 years [IQR, 57–79], and 31 435 pairs (45.6%) were from female patients. ICU‐level care at the time of echocardiography was present in 19 331 pairs (28.1%). Common comorbidities included hypertension in 41 369 pairs (60.0%), heart failure in 24 900 pairs (36.1%), coronary artery disease in 26 124 pairs (37.9%), atrial fibrillation in 20 777 pairs (30.2%), chronic kidney disease in 16 183 pairs (23.5%), and chronic obstructive pulmonary disease in 8245 pairs (12.0%) (Table 1).

**TABLE 1:** Baseline characteristics by machine‐reported RAD/RVH status.

Characteristic	Overall	RAD/RVH negative	RAD/RVH positive
*N*	68 905	66 090	2815
Age, median [IQR]	68 [57–79]	68 [57–79]	67 [56–78]
Female	31 435 (45.6%)	30 144 (45.6%)	1291 (45.9%)
ICU at echo	19 331 (28.1%)	18 252 (27.6%)	1079 (38.3%)
Hypertension	41 369 (60.0%)	39 691 (60.1%)	1678 (59.6%)
Diabetes	19 985 (29.0%)	19 033 (28.8%)	952 (33.8%)
Heart failure	24 900 (36.1%)	23 288 (35.2%)	1612 (57.3%)
COPD	8245 (12.0%)	7711 (11.7%)	534 (19.0%)
Chronic kidney disease	16 183 (23.5%)	15 337 (23.2%)	846 (30.1%)
Atrial fibrillation	20 777 (30.2%)	19 591 (29.6%)	1186 (42.1%)
Coronary artery disease	26 124 (37.9%)	24 882 (37.6%)	1242 (44.1%)
Pulmonary embolism	2479 (3.6%)	2360 (3.6%)	119 (4.2%)
Echo‐PH primary	25 268 (36.7%)	23 808 (36.0%)	1460 (51.9%)
Echo‐PH specific	13 321 (19.3%)	12 322 (18.6%)	999 (35.5%)
Echo RV abnormality	33 525 (48.7%)	31 665 (47.9%)	1860 (66.1%)
RV dysfunction	11 178 (16.2%)	10 096 (15.3%)	1082 (38.4%)
RV dilation	9315 (13.5%)	8506 (12.9%)	809 (28.7%)
RA enlargement	21 021 (30.5%)	19 996 (30.3%)	1025 (36.4%)
Moderate/severe TR	14 298 (20.8%)	13 259 (20.1%)	1039 (36.9%)

*Note*: Values are shown at the ECG–echocardiography pair level.

Abbreviations: COPD, chronic obstructive pulmonary disease; ECG, electrocardiogram; ICU, intensive care unit; IQR, interquartile range; PH, pulmonary hypertension; RA, right atrial; RAD, right axis deviation; RV, right ventricular; RVH, right ventricular hypertrophy; TR, tricuspid regurgitation.

Patients with machine‐reported RAD/RVH had a higher burden of cardiopulmonary disease than those without RAD/RVH. ICU status at echocardiography was more common in the RAD/RVH‐positive group than in the RAD/RVH‐negative group (38.3% vs. 27.6%). Heart failure (57.3% vs. 35.2%), chronic obstructive pulmonary disease (19.0% vs. 11.7%), chronic kidney disease (30.1% vs. 23.2%), atrial fibrillation (42.1% vs. 29.6%), and coronary artery disease (44.1% vs. 37.6%) were also more frequent among RAD/RVH‐positive pairs (Table 1).

### 3.2 Echocardiographic Findings by RAD/RVH Status

Echocardiographic abnormalities were more frequent among ECG–echocardiography pairs with machine‐reported RAD/RVH. Echo‐PH specific was present in 999 of 2815 RAD/RVH‐positive pairs (35.5%) compared with 12 322 of 66 090 RAD/RVH‐negative pairs (18.6%). Echo‐PH primary was present in 51.9% of RAD/RVH‐positive pairs compared with 36.0% of RAD/RVH‐negative pairs. Echo RV abnormality was present in 66.1% of RAD/RVH‐positive pairs compared with 47.9% of RAD/RVH‐negative pairs (Table 1).

Individual right‐heart echocardiographic abnormalities also occurred more frequently in the RAD/RVH‐positive group. RV dysfunction was present in 38.4% of RAD/RVH‐positive pairs versus 15.3% of RAD/RVH‐negative pairs, RV dilation in 28.7% versus 12.9%, and moderate or severe tricuspid regurgitation in 36.9% versus 20.1% (Table 1).

### 3.3 ECG–Echocardiography Interval and Tricuspid Regurgitation Velocity Availability

The median absolute ECG–echocardiography interval was 19.7 h [IQR, 5.6–45.1], and 40 036 pairs (58.1%) were obtained within 24 h. Tricuspid regurgitation velocity was available in 45 273 pairs and missing in 23 632 pairs (34.3%). Additional details on ECG–echocardiography timing and tricuspid regurgitation velocity availability are shown in Table S8.

### 3.4 Diagnostic Performance of Machine‐Reported ECG Findings

Machine‐reported RAD/RVH showed low sensitivity but high specificity for echocardiographic outcomes. For Echo‐PH specific, RAD/RVH had sensitivity 7.5% (95% CI, 7.1–8.0), specificity 96.7% (95% CI, 96.6–96.9), positive predictive value 35.5% (95% CI, 33.7–37.3), negative predictive value 81.4% (95% CI, 81.1–81.7), positive likelihood ratio 2.30 (95% CI, 2.13–2.47), and negative likelihood ratio 0.96 (95% CI, 0.95–0.96). Diagnostic performance for additional ECG findings and outcomes is shown in Table 2 and Figure S1.

**TABLE 2:** Diagnostic performance of ECG findings for echocardiographic outcomes.

ECG finding	Echocardiographic outcome	*N*	TP	FP	FN	TN	Sensitivity (%) (95% CI)	Specificity (%) (95% CI)	PPV (%) (95% CI)	NPV (%) (95% CI)	LR+ (95% CI)	LR− (95% CI)
RAD/RVH	Echo‐PH specific	68 905	999	1816	12 322	53 768	7.5 (7.1–8.0)	96.7 (96.6–96.9)	35.5 (33.7–37.3)	81.4 (81.1–81.7)	2.30 (2.13–2.47)	0.96 (0.95–0.96)
RAD	Echo‐PH specific	68 905	852	1493	12 469	54 091	6.4 (6.0–6.8)	97.3 (97.2–97.4)	36.3 (34.4–38.3)	81.3 (81.0–81.6)	2.38 (2.19–2.58)	0.96 (0.96–0.97)
RVH	Echo‐PH specific	68 905	316	467	13 005	55 117	2.4 (2.1–2.6)	99.2 (99.1–99.2)	40.4 (36.9–43.9)	80.9 (80.6–81.2)	2.82 (2.45–3.25)	0.98 (0.98–0.99)
QRS axis >110°	Echo‐PH specific	68 905	655	1109	12 666	54 475	4.9 (4.6–5.3)	98.0 (97.9–98.1)	37.1 (34.9–39.4)	81.1 (80.8–81.4)	2.46 (2.24–2.71)	0.97 (0.97–0.97)
RBBB	Echo‐PH specific	68 905	1699	5018	11 622	50 566	12.8 (12.2–13.3)	91.0 (90.7–91.2)	25.3 (24.3–26.4)	81.3 (81.0–81.6)	1.41 (1.34–1.49)	0.96 (0.95–0.97)
RAD/RVH	Echo‐PH primary	68 905	1460	1355	23 808	42 282	5.8 (5.5–6.1)	96.9 (96.7–97.1)	51.9 (50.0–53.7)	64.0 (63.6–64.3)	1.86 (1.73–2.00)	0.97 (0.97–0.98)
RAD	Echo‐PH primary	68 905	1226	1119	24 042	42 518	4.9 (4.6–5.1)	97.4 (97.3–97.6)	52.3 (50.2–54.3)	63.9 (63.5–64.2)	1.89 (1.75–2.05)	0.98 (0.97–0.98)
RVH	Echo‐PH primary	68 905	434	349	24 834	43 288	1.7 (1.6–1.9)	99.2 (99.1–99.3)	55.4 (51.9–58.9)	63.5 (63.2–63.9)	2.15 (1.87–2.47)	0.99 (0.99–0.99)
QRS axis >110°	Echo‐PH primary	68 905	958	806	24 310	42 831	3.8 (3.6–4.0)	98.2 (98.0–98.3)	54.3 (51.9–56.7)	63.8 (63.4–64.2)	2.05 (1.87–2.25)	0.98 (0.98–0.98)
RBBB	Echo‐PH primary	68 905	2961	3756	22 307	39 881	11.7 (11.3–12.1)	91.4 (91.1–91.7)	44.1 (42.9–45.3)	64.1 (63.8–64.5)	1.36 (1.30–1.42)	0.97 (0.96–0.97)
RAD/RVH	Echo RV abnormality	68 905	1860	955	31 665	34 425	5.5 (5.3–5.8)	97.3 (97.1–97.5)	66.1 (64.3–67.8)	52.1 (51.7–52.5)	2.06 (1.90–2.22)	0.97 (0.97–0.97)
RAD	Echo RV abnormality	68 905	1569	776	31 956	34 604	4.7 (4.5–4.9)	97.8 (97.6–98.0)	66.9 (65.0–68.8)	52.0 (51.6–52.4)	2.13 (1.96–2.32)	0.97 (0.97–0.98)
RVH	Echo RV abnormality	68 905	543	240	32 982	35 140	1.6 (1.5–1.8)	99.3 (99.2–99.4)	69.3 (66.0–72.6)	51.6 (51.2–52.0)	2.39 (2.05–2.78)	0.99 (0.99–0.99)
QRS axis >110°	Echo RV abnormality	68 905	1241	523	32 284	34 857	3.7 (3.5–3.9)	98.5 (98.4–98.6)	70.4 (68.2–72.5)	51.9 (51.5–52.3)	2.50 (2.26–2.77)	0.98 (0.98–0.98)
RBBB	Echo RV abnormality	68 905	4054	2663	29 471	32 717	12.1 (11.7–12.4)	92.5 (92.2–92.7)	60.4 (59.2–61.5)	52.6 (52.2–53.0)	1.61 (1.53–1.68)	0.95 (0.95–0.96)

*Note*: Diagnostic performance was calculated at the ECG–echocardiography pair level.

Abbreviations: CI, confidence interval; ECG, electrocardiogram; FN, false negative; FP, false positive; LR−, negative likelihood ratio; LR+, positive likelihood ratio; NPV, negative predictive value; PH, pulmonary hypertension; PPV, positive predictive value; RAD, right axis deviation; RBBB, right bundle branch block; RV, right ventricular; RVH, right ventricular hypertrophy; TN, true negative; TP, true positive.

Overall, machine‐reported RAD/RVH, RAD, RVH, and QRS axis greater than 110° were highly specific but insensitive for echocardiographic pulmonary hypertension phenotypes and right‐heart abnormalities. Right bundle branch block had higher sensitivity than RAD/RVH but lower specificity.

### 3.5 Adjusted Associations

**FIGURE 2:**
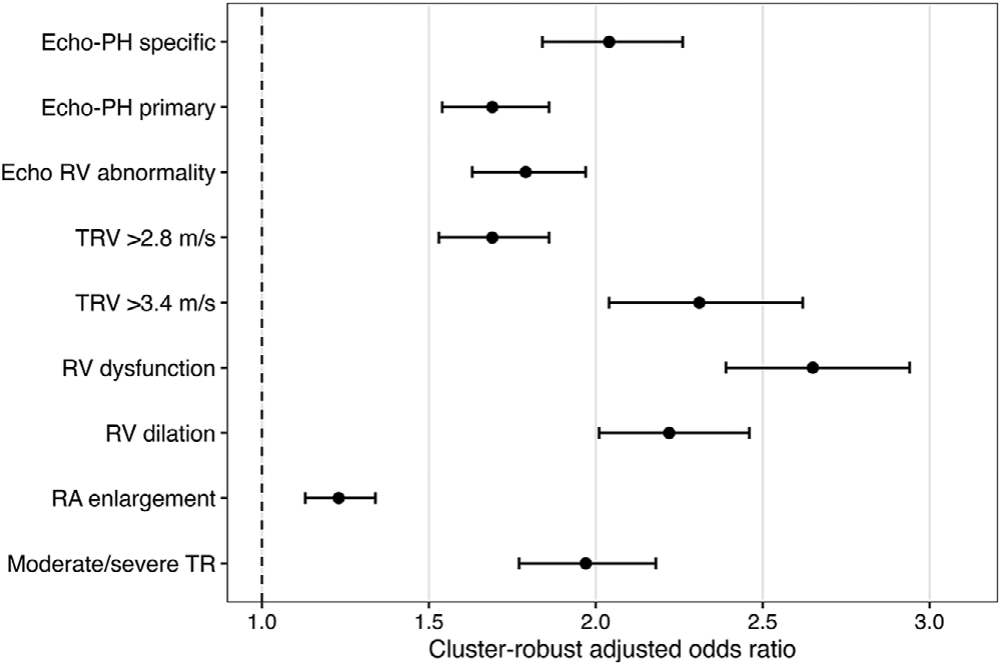
Cluster‐robust adjusted associations between machine‐reported RAD/RVH and echocardiographic outcomes. Forest plot showing patient‐level cluster‐robust adjusted odds ratios and 95% confidence intervals for the association between machine‐reported RAD/RVH and echocardiographic pulmonary hypertension phenotypes and right‐heart abnormalities. Models adjusted for age, sex, race, hypertension, diabetes mellitus, heart failure, chronic obstructive pulmonary disease, chronic kidney disease, atrial fibrillation, coronary artery disease, pulmonary embolism, and ICU status at echocardiography. ICU, intensive care unit; RAD, right axis deviation; RVH, right ventricular hypertrophy; TRV, tricuspid regurgitation velocity.

After accounting for repeated ECG–echocardiography pairs within patients using cluster‐robust standard errors, machine‐reported RAD/RVH remained associated with Echo‐PH specific (adjusted OR, 2.04; 95% CI, 1.84–2.26; *p* < 0.001) (Table 3; Figure 2). Associations were also observed for Echo‐PH primary (adjusted OR, 1.69; 95% CI, 1.54–1.86), Echo RV abnormality (adjusted OR, 1.79; 95% CI, 1.63–1.97), tricuspid regurgitation velocity greater than 2.8 m/s in the full analytic cohort (adjusted OR, 1.69; 95% CI, 1.53–1.86), and tricuspid regurgitation velocity greater than 3.4 m/s in the full analytic cohort (adjusted OR, 2.31; 95% CI, 2.04–2.62).

**TABLE 3:** Cluster‐robust adjusted associations between machine‐reported RAD/RVH and echocardiographic outcomes.

Outcome	ECG–echo pairs	Patients	Events	RAD/RVH‐positive pairs	Cluster‐robust adjusted OR (95% CI)	*p* value
Echo‐PH specific	68 905	42 078	13 321	2815	2.04 (1.84–2.26)	<0.001
Echo‐PH primary	68 905	42 078	25 268	2815	1.69 (1.54–1.86)	<0.001
Echo RV abnormality	68 905	42 078	33 525	2815	1.79 (1.63–1.97)	<0.001
TR velocity >2.8 m/s	68 905	42 078	17 409	2815	1.69 (1.53–1.86)	<0.001
TR velocity >3.4 m/s	68 905	42 078	6319	2815	2.31 (2.04–2.62)	<0.001
RV dysfunction	68 905	42 078	11 178	2815	2.65 (2.39–2.94)	<0.001
RV dilation	68 905	42 078	9315	2815	2.22 (2.01–2.46)	<0.001
RA enlargement	68 905	42 078	21 021	2815	1.23 (1.13–1.34)	<0.001
Moderate/severe TR	68 905	42 078	14 298	2815	1.97 (1.77–2.18)	<0.001

*Note*: Odds ratios were estimated using fully adjusted logistic regression models with patient‐level cluster‐robust standard errors. Models were adjusted for age, sex, race, hypertension, diabetes mellitus, heart failure, chronic obstructive pulmonary disease, chronic kidney disease, atrial fibrillation, coronary artery disease, pulmonary embolism, and ICU status at echocardiography.

Abbreviations: CI, confidence interval; ECG, electrocardiogram; ICU, intensive care unit; OR, odds ratio; PH, pulmonary hypertension; RA, right atrial; RAD, right axis deviation; RV, right ventricular; RVH, right ventricular hypertrophy; TR, tricuspid regurgitation.

Component outcomes showed similar associations, including RV dysfunction (adjusted OR, 2.65; 95% CI, 2.39–2.94), RV dilation (adjusted OR, 2.22; 95% CI, 2.01–2.46), RA enlargement (adjusted OR, 1.23; 95% CI, 1.13–1.34), and moderate or severe tricuspid regurgitation (adjusted OR, 1.97; 95% CI, 1.77–2.18). These results are shown in Table 3. Diagnostic performance for component echocardiographic outcomes is shown in Table S5.

For Echo‐PH specific, the adjusted probability was 18.8% (95% CI, 18.4–19.2) without RAD/RVH and 30.0% (95% CI, 28.3–31.7) with RAD/RVH, corresponding to an adjusted risk difference of 11.2% points (95% CI, 9.5–12.9) and an adjusted risk ratio of 1.59 (95% CI, 1.50–1.69). Adjusted marginal probabilities, risk differences, and risk ratios for all outcomes are shown in Table S4.

### 3.6 Sensitivity Analyses

The association between RAD/RVH and echocardiographic outcomes remained robust in patient‐level sensitivity analyses restricted to one ECG–echocardiography pair per patient. When the first echocardiogram per patient was used, RAD/RVH remained associated with Echo‐PH specific in the fully adjusted model (OR, 2.05; 95% CI, 1.80–2.34). When the ECG–echocardiography pair with the closest time interval per patient was used, the corresponding adjusted odds ratio was 2.09 (95% CI, 1.84–2.37). Similar findings were observed for Echo‐PH primary and Echo RV abnormality (Table S1 and Figure S3).

Results were also consistent across alternative ECG–echocardiography matching windows. For Echo‐PH specific, the unadjusted odds ratio for RAD/RVH was 2.40 (95% CI, 2.22–2.60) in the primary pre‐echo 7‐day window, 2.19 (95% CI, 2.00–2.39) using ECGs within plus or minus 1 day, 2.32 (95% CI, 2.15–2.51) using ECGs within plus or minus 3 days, and 2.42 (95% CI, 2.25–2.61) using ECGs within plus or minus 7 days. Findings were similarly stable for Echo‐PH primary and Echo RV abnormality (Table S2 and Figure S2).

Among ECG–echocardiography pairs with available tricuspid regurgitation velocity, the analytic cohort included 45 273 pairs from 32 042 patients. RAD/RVH was present in 1894 pairs. For tricuspid regurgitation velocity greater than 2.8 m/s, RAD/RVH had sensitivity 6.3%, specificity 97.1%, positive predictive value 57.9%, and negative predictive value 62.4%. For tricuspid regurgitation velocity greater than 3.4 m/s, RAD/RVH had sensitivity 9.1%, specificity 96.6%, positive predictive value 30.5%, and negative predictive value 86.8% (Table S3a). In fully adjusted models, RAD/RVH remained associated with tricuspid regurgitation velocity greater than 2.8 m/s (OR, 1.89; 95% CI, 1.70–2.09) and tricuspid regurgitation velocity greater than 3.4 m/s (OR, 2.39; 95% CI, 2.14–2.67) (Table S3b and Figure S3).

### 3.7 ECG Text Extraction Validation

In the validation sample of 200 ECG reports, the automated RAD/RVH extraction showed a positive predictive value of 91.0% (95% CI, 83.6–95.8), negative predictive value of 100.0% (95% CI, 96.4–100.0), and accuracy of 95.5% (95% CI, 91.6–97.9). The nine discordant cases were automated RVH‐positive reports that contained uncertain wording such as possible, probable, or consider RVH and were not adjudicated as definite RVH. Detailed validation results and discordant cases are shown in Tables S6 and S7.

## 4 Discussion

### 4.1 Principal Findings

In this large linked ECG–echocardiography cohort, machine‐reported RAD/RVH on routine ECG was associated with echo‐derived pulmonary hypertension phenotypes and right‐heart abnormalities in hospitalized patients after adjustment for demographics, comorbidities, ICU status, and repeated ECG–echocardiography pairs within patients. However, the diagnostic profile was characterized by low sensitivity and high specificity. These findings suggest that machine‐reported RAD/RVH should be interpreted as a specific but low‐sensitivity supportive clue for echocardiographic right‐heart disease, rather than as a standalone screening test or definitive diagnostic marker.

The addition of patient‐level cluster‐robust standard errors did not materially change the primary association. Machine‐reported RAD/RVH remained associated with Echo‐PH specific, and similar associations were observed across broader pulmonary hypertension phenotypes, RV abnormality, tricuspid regurgitation velocity thresholds, RV dysfunction, RV dilation, and moderate or severe tricuspid regurgitation. The adjusted marginal probability analysis further supported the clinical interpretability of this association: the adjusted probability of Echo‐PH specific was 18.8% in the absence of RAD/RVH and 30.0% in its presence, corresponding to an absolute risk difference of 11.2% points. This absolute difference is more clinically interpretable than the odds ratio alone, particularly because several echocardiographic outcomes were common in the study population.

At the same time, the low sensitivity indicates that the absence of machine‐reported RAD/RVH cannot exclude echo‐derived pulmonary hypertension phenotypes or right‐heart abnormalities. This is consistent with the known limitations of routine ECG findings for detecting right‐sided pressure or structural abnormalities. In clinical practice, these findings may therefore be most useful when present: they can prompt closer attention to the right heart on echocardiography or support suspicion of right‐heart disease in the appropriate clinical context. They should not be used to defer echocardiographic evaluation when clinical suspicion remains.

### 4.2 Comparison With Prior ECG Studies

These findings are consistent with prior studies showing that conventional ECG markers of right‐heart strain tend to have high specificity but limited sensitivity for pulmonary hypertension or right ventricular remodeling. Al‐Naamani et al. reported that ECG findings such as RAD and QRS axis greater than 110° were associated with pulmonary hypertension, but that absence of ECG criteria could not exclude the disease [17]. Similarly, data from the Pan‐African Pulmonary Hypertension Cohort showed that ECG abnormalities related to right‐heart strain had variable but generally limited sensitivity and relatively high specificity [18]. Earlier work also emphasized the limited role of the electrocardiogram as a standalone test for pulmonary hypertension [19]. A recent diagnostic accuracy study further supports the concept that selected ECG‐derived right‐heart criteria may help identify pulmonary hypertension but do not provide sufficient sensitivity to exclude disease [25].

Several prior studies have evaluated ECG criteria against imaging‐defined RVH or dilation. Kopeć et al. showed that ECG criteria were associated with RVH and dilation in idiopathic pulmonary arterial hypertension, but sensitivity remained limited [20]. The MESA‐Right Ventricle Study similarly demonstrated that surface ECG criteria for RVH have limited validity when compared with cardiac magnetic resonance imaging [21]. Other studies comparing ECG criteria with imaging‐defined right ventricular remodeling have reached similar conclusions [22, 23, 24]. This study extends that literature by evaluating routinely generated machine‐reported ECG text rather than manually applied ECG criteria, and by linking these findings to structured echocardiographic phenotypes in a large real‐world hospitalized cohort.

### 4.3 Relevance to Echocardiographic Right‐Heart Assessment

The present findings are particularly relevant to echocardiographic practice because the outcomes were based on structured echocardiographic findings rather than solely on diagnostic labels. Current echocardiographic guidelines emphasize comprehensive right‐heart assessment, including tricuspid regurgitation velocity, right ventricular size and function, right atrial size, inferior vena cava findings, and tricuspid regurgitation severity [5, 7, 8, 10, 11]. The composite outcome of Echo RV abnormality was designed to reflect this broader right‐heart phenotype, and RAD/RVH was associated not only with Echo‐PH specific but also with Echo RV abnormality.

The tricuspid regurgitation velocity‐only analysis further strengthens the interpretation of the main findings. Composite echocardiographic outcomes may be influenced by how structured text findings are recorded, so tricuspid regurgitation velocity thresholds were evaluated separately. RAD/RVH remained independently associated with both tricuspid regurgitation velocity greater than 2.8 m/s and greater than 3.4 m/s, with stronger association for the higher threshold. This supports the view that the observed associations were not driven solely by text‐based echo labels, but also by Doppler‐derived evidence of elevated pulmonary pressure.

Large echocardiographic cohort studies have shown that even mild elevations in estimated pulmonary pressures are associated with adverse outcomes and right ventricular dysfunction [12, 13]. In this context, a highly specific ECG signal may be clinically useful even if insensitive. The value of RAD/RVH is not that it identifies most patients with echo‐derived pulmonary hypertension, but that its presence should increase suspicion for relevant right‐heart abnormalities and may guide more careful interpretation of echocardiographic right‐heart parameters [14, 15, 16].

### 4.4 Relationship to ECG Waveform‐Based Prediction Studies

Recent waveform‐based ECG prediction studies have demonstrated that raw ECG waveforms can identify pulmonary hypertension or elevated pulmonary arterial pressure with promising discrimination [26, 27, 28, 29, 30]. These studies suggest that ECGs contain more information about pulmonary vascular and right‐heart disease than is captured by conventional visual or rule‐based criteria. However, such models require dedicated algorithm development, validation, and clinical implementation. In contrast, machine‐generated ECG interpretation text is already routinely available in many clinical workflows.

This study therefore addresses a different and more pragmatic question: what is the clinical meaning of right‐heart findings already reported by routine ECG machines? The answer appears to be that machine‐reported RAD/RVH is a specific but insensitive supportive clue for echocardiographic pulmonary hypertension phenotypes and right‐heart abnormalities. Thus, these findings complement rather than compete with ECG waveform‐based prediction studies. Dedicated ECG prediction models may ultimately provide higher sensitivity and discrimination, whereas routine machine‐reported text may provide an immediately available but limited signal that can inform clinical interpretation.

### 4.5 ECG Text Extraction Validation

The validation analysis supported the reliability of the automated ECG text extraction for the primary composite exposure. In a random sample of 200 ECG reports, the automated RAD/RVH extraction had a positive predictive value of 91.0%, negative predictive value of 100.0%, and accuracy of 95.5%. The discordant cases were limited to automated RVH‐positive reports with uncertain wording, such as possible, probable, or consider RVH. This finding clarifies that the main source of misclassification was not RAD extraction, but uncertain RVH wording. Because uncertain RVH statements were not adjudicated as definite positive findings in the validation review, the analytic exposure may include a small number of less definite RVH reports. This limitation is unlikely to fully explain the observed associations but supports cautious interpretation of the RVH component.

### 4.6 Clinical Implications

The low sensitivity observed in this study means that the absence of machine‐reported RAD/RVH should not reassure clinicians that echocardiographic pulmonary hypertension phenotypes or right‐heart abnormalities are absent. Most patients with Echo‐PH specific did not have RAD/RVH on ECG. This is consistent with prior ECG literature and reinforces the limited role of conventional ECG findings as screening tests.

Conversely, the high specificity and consistent adjusted associations suggest that the presence of machine‐reported RAD/RVH should not be ignored. In a hospitalized patient with RAD or RVH on routine ECG, clinicians should consider whether there is corresponding evidence of elevated pulmonary pressure, RV dysfunction, RV dilation, RA enlargement, or significant tricuspid regurgitation on echocardiography. The finding may be particularly useful as a low‐cost contextual clue when interpreting echocardiograms or deciding whether right‐heart abnormalities deserve closer attention in the clinical record.

Importantly, these results do not imply that RAD/RVH should be used to diagnose pulmonary hypertension. Echocardiography itself is not the definitive diagnostic standard for pulmonary hypertension, and right heart catheterization remains required for hemodynamic confirmation [5, 6]. Rather, machine‐reported RAD/RVH should be viewed as a specific but low‐sensitivity supportive clue associated with echocardiographic right‐heart phenotypes in real‐world hospitalized patients.

### 4.7 Strengths and Limitations

This study has several strengths. First, it used a large linked ECG–echocardiography cohort with 68 905 ECG–echocardiography pairs from 42 078 patients, providing substantially greater power than many prior ECG and pulmonary hypertension studies. Second, the exposure was based on routine machine‐reported ECG text, making the findings directly relevant to real‐world clinical workflows. Third, outcomes incorporated structured echocardiographic findings, including Echo‐PH specific, broader Echo‐PH, Echo RV abnormality, tricuspid regurgitation velocity‐only thresholds, and component right‐heart abnormalities. Fourth, the main findings were consistent across several sensitivity analyses, including patient‐level restriction, alternative ECG–echocardiography time windows, and tricuspid regurgitation velocity‐only outcomes. Fifth, the revision analyses accounted for repeated ECG–echocardiography pairs within patients using cluster‐robust standard errors and supplemented odds ratios with adjusted marginal probabilities, risk differences, and risk ratios. Finally, the ECG text extraction was evaluated in a validation sample using predefined adjudication rules.

This study also has important limitations. First, pulmonary hypertension was defined using echocardiographic findings rather than right heart catheterization. Therefore, the outcomes should be interpreted as echo‐derived pulmonary hypertension phenotypes or echocardiographic pulmonary hypertension phenotypes, not as confirmed hemodynamic pulmonary hypertension. Second, the study was based on deidentified data from a single health system represented in MIMIC‐IV, and findings may not generalize to other institutions, ECG vendors, machine interpretation algorithms, or patient populations. External validation in other ECG–echocardiography datasets would be valuable.

Third, the ECG exposure was based on machine‐generated report text rather than manual ECG adjudication or raw waveform analysis. Machine‐reported interpretations may vary by algorithm and may contain false positives or false negatives. The validation analysis showed good overall agreement for the composite RAD/RVH exposure, but uncertain RVH wording contributed to a small number of discordant cases. Fourth, structured echocardiographic measurements and categorical findings may be incomplete or inconsistently recorded. To address this, multiple outcome definitions were evaluated, and tricuspid regurgitation velocity‐only analyses were performed among studies with available tricuspid regurgitation velocity. Tricuspid regurgitation velocity was missing in 34.3% of ECG–echocardiography pairs, so tricuspid regurgitation velocity‐only outcomes should be interpreted in the context of this availability.

Fifth, residual confounding by cardiopulmonary disease severity may remain despite adjustment for major comorbidities and ICU status. Additional markers of acute illness severity, such as oxygen requirement, mechanical ventilation, vasopressor use, and laboratory values, were not incorporated into the primary model and should be considered in future validation studies. Finally, because this was an observational study, the associations should not be interpreted causally. ECG findings and echocardiographic abnormalities may both reflect underlying cardiopulmonary disease burden.

## 5 Conclusions

In a large linked MIMIC‐IV ECG–echocardiography cohort, machine‐reported ECG RAD or RVH was associated with echocardiographic pulmonary hypertension phenotypes and right‐heart abnormalities after adjustment and after accounting for repeated observations within patients. These findings were highly specific but insensitive, indicating that routine machine‐reported ECG right‐heart findings may serve as supportive clues but should not be used to exclude echocardiographic pulmonary hypertension phenotypes or right‐heart disease. The association persisted in patient‐level, time‐window, and tricuspid regurgitation velocity‐only sensitivity analyses, supporting the clinical relevance of routine ECG machine interpretation as a pragmatic signal of echocardiographic right‐heart abnormality.

## Funding

No specific funding was received for this study.

## Ethics Statement

This study used deidentified data available through PhysioNet under credentialed access. The analysis was conducted in accordance with the applicable data use agreements. Because the study used deidentified secondary data, additional institutional review board approval was not required.

## Conflicts of Interest

The author declares no conflicts of interest.

## Code Availability Statement

Analytic code can be made available from the author upon reasonable request, subject to the data use requirements of PhysioNet.

## Supporting information




**Supplementary Table 1**: Patient‐level sensitivity analysis.
**Supplementary Table 2**: Time‐window sensitivity analysis.
**Supplementary Table 3a** TR velocity‐only diagnostic performance.
**Supplementary Table 3b**: TR velocity‐only adjusted models.
**Supplementary Table 4**: Adjusted marginal probabilities, risk differences, and risk ratios.
**Supplementary Table 5**: Diagnostic performance of machine‐reported RAD/RVH for component right‐heart outcomes.
**Supplementary Table 6**: Validation of automated ECG text extraction.
**Supplementary Table 7**: Discordant cases in ECG text extraction validation.
**Supplementary Table 8**: ECGechocardiography interval and TR velocity availability.
**Supplementary Figure 1**: Diagnostic performance of ECG findings for selected echocardiographic outcomes. Diagnostic performance of machine‐reported ECG findings is shown for Echo‐PH specific and Echo RV abnormality. Performance metrics include sensitivity, specificity, positive predictive value, and negative predictive value.
**Supplementary Figure 2**: Time‐window sensitivity analysis. Unadjusted odds ratios and 95% confidence intervals are shown for machine‐reported RAD/RVH across alternative ECGechocardiography matching windows for selected echocardiographic outcomes.
**Supplementary Figure 3**: Patient‐level and TR velocity‐only sensitivity analyses. Adjusted odds ratios and 95% confidence intervals are shown for patient‐level sensitivity analyses and TR velocity‐only sensitivity analyses.

## Data Availability

The data that support the findings of this study are available from PhysioNet under credentialed access and applicable data use agreements. The datasets include MIMIC‐IV, MIMIC‐IV‐ECG, and MIMIC‐IV‐ECHO. MIMIC‐IV‐ECG and MIMIC‐IV‐ECHO are available at https://physionet.org/content/mimic‐iv‐ecg/1.0/ and https://physionet.org/content/mimic‐iv‐echo/1.0/, with DOIs 10.13026/4nqg‐sb35 and 10.13026/nrjh‐5r77, respectively. These data are not redistributed with this manuscript because users must complete the required training, credentialing, and data use agreement through PhysioNet.
