# Supplementary material for: Machine‐Reported Electrocardiographic Right Axis Deviation or Right Ventricular Hypertrophy and Echocardiographic Pulmonary Hypertension Phenotypes and Right‐Heart Abnormalities
Source: Echocardiography. 2026 Jul 13;43(7):e70539. doi: 10.1111/echo.70539 (PMC13358884; doi:10.1111/echo.70539)

**Supplementary Material**

**Machine-Reported Electrocardiographic Right Axis Deviation or Right Ventricular Hypertrophy and Echocardiographic Pulmonary Hypertension Phenotypes and Right-Heart Abnormalities**

**Author:** Yoshihiro Fuchigami

**Supplementary Table 1. Patient-level sensitivity analysis**

| **Dataset** | **Outcome** | **Model** | **OR (95% CI)** | **P value** | **N used** | **Patients** | **Exposure positive** | **Outcome positive** |
| --- | --- | --- | --- | --- | --- | --- | --- | --- |
| Closest ECG–echo per patient | Echo-PH specific | Model 1: unadjusted | 2.48 (2.21–2.77) | <0.001 | 42,078 | 42,078 | 1,443 | 7,303 |
| Closest ECG–echo per patient | Echo-PH specific | Model 2: age + sex + race | 2.72 (2.41–3.07) | <0.001 | 42,077 | 42,078 | 1,443 | 7,303 |
| Closest ECG–echo per patient | Echo-PH specific | Model 3: demographics + comorbidities + ICU | 2.09 (1.84–2.37) | <0.001 | 42,077 | 42,078 | 1,443 | 7,303 |
| Closest ECG–echo per patient | Echo-PH primary | Model 1: unadjusted | 2.02 (1.82–2.25) | <0.001 | 42,078 | 42,078 | 1,443 | 14,590 |
| Closest ECG–echo per patient | Echo-PH primary | Model 2: age + sex + race | 2.25 (2.01–2.52) | <0.001 | 42,077 | 42,078 | 1,443 | 14,590 |
| Closest ECG–echo per patient | Echo-PH primary | Model 3: demographics + comorbidities + ICU | 1.79 (1.59–2.01) | <0.001 | 42,077 | 42,078 | 1,443 | 14,590 |
| Closest ECG–echo per patient | Echo RV abnormality | Model 1: unadjusted | 2.10 (1.88–2.34) | <0.001 | 42,078 | 42,078 | 1,443 | 19,456 |
| Closest ECG–echo per patient | Echo RV abnormality | Model 2: age + sex + race | 2.22 (1.99–2.49) | <0.001 | 42,077 | 42,078 | 1,443 | 19,456 |
| Closest ECG–echo per patient | Echo RV abnormality | Model 3: demographics + comorbidities + ICU | 1.79 (1.59–2.01) | <0.001 | 42,077 | 42,078 | 1,443 | 19,456 |
| First echo per patient | Echo-PH specific | Model 1: unadjusted | 2.45 (2.18–2.75) | <0.001 | 42,078 | 42,078 | 1,359 | 7,275 |
| First echo per patient | Echo-PH specific | Model 2: age + sex + race | 2.69 (2.38–3.04) | <0.001 | 42,077 | 42,078 | 1,359 | 7,275 |
| First echo per patient | Echo-PH specific | Model 3: demographics + comorbidities + ICU | 2.05 (1.80–2.34) | <0.001 | 42,077 | 42,078 | 1,359 | 7,275 |
| First echo per patient | Echo-PH primary | Model 1: unadjusted | 2.03 (1.82–2.26) | <0.001 | 42,078 | 42,078 | 1,359 | 14,710 |
| First echo per patient | Echo-PH primary | Model 2: age + sex + race | 2.27 (2.02–2.56) | <0.001 | 42,077 | 42,078 | 1,359 | 14,710 |
| First echo per patient | Echo-PH primary | Model 3: demographics + comorbidities + ICU | 1.80 (1.59–2.03) | <0.001 | 42,077 | 42,078 | 1,359 | 14,710 |
| First echo per patient | Echo RV abnormality | Model 1: unadjusted | 2.05 (1.84–2.30) | <0.001 | 42,078 | 42,078 | 1,359 | 19,511 |
| First echo per patient | Echo RV abnormality | Model 2: age + sex + race | 2.16 (1.93–2.43) | <0.001 | 42,077 | 42,078 | 1,359 | 19,511 |
| First echo per patient | Echo RV abnormality | Model 3: demographics + comorbidities + ICU | 1.75 (1.55–1.98) | <0.001 | 42,077 | 42,078 | 1,359 | 19,511 |

Patient-level sensitivity analyses restricted the analytic dataset to one ECG–echocardiography pair per patient.

**Supplementary Table 2. Time-window sensitivity analysis**

| **Dataset** | **Outcome** | **N** | **Patients** | **Exposure positive** | **Outcome positive** | **Sensitivity** | **Specificity** | **PPV** | **NPV** | **OR (95% CI)** | **P value** |
| --- | --- | --- | --- | --- | --- | --- | --- | --- | --- | --- | --- |
| Pre-echo ECG within 7 days | Echo-PH specific | 68,905 | 42,078 | 2,815 | 13,321 | 7.5 | 96.7 | 35.5 | 81.4 | 2.40 (2.22–2.60) | <0.001 |
| Pre-echo ECG within 7 days | Echo-PH primary | 68,905 | 42,078 | 2,815 | 25,268 | 5.8 | 96.9 | 51.9 | 64.0 | 1.91 (1.77–2.06) | <0.001 |
| Pre-echo ECG within 7 days | Echo RV abnormality | 68,905 | 42,078 | 2,815 | 33,525 | 5.5 | 97.3 | 66.1 | 52.1 | 2.12 (1.96–2.29) | <0.001 |
| ECG within ±1 day | Echo-PH specific | 51,870 | 32,721 | 2,190 | 10,191 | 7.3 | 96.5 | 33.9 | 81.0 | 2.19 (2.00–2.39) | <0.001 |
| ECG within ±1 day | Echo-PH primary | 51,870 | 32,721 | 2,190 | 19,201 | 5.7 | 96.7 | 50.3 | 63.6 | 1.77 (1.62–1.93) | <0.001 |
| ECG within ±1 day | Echo RV abnormality | 51,870 | 32,721 | 2,190 | 26,265 | 5.5 | 97.1 | 66.0 | 50.0 | 1.94 (1.78–2.13) | <0.001 |
| ECG within ±3 days | Echo-PH specific | 70,643 | 41,492 | 2,917 | 13,817 | 7.4 | 96.7 | 35.1 | 81.1 | 2.32 (2.15–2.51) | <0.001 |
| ECG within ±3 days | Echo-PH primary | 70,643 | 41,492 | 2,917 | 26,301 | 5.7 | 96.8 | 51.5 | 63.4 | 1.84 (1.71–1.98) | <0.001 |
| ECG within ±3 days | Echo RV abnormality | 70,643 | 41,492 | 2,917 | 35,306 | 5.5 | 97.2 | 66.5 | 50.7 | 2.04 (1.89–2.21) | <0.001 |
| ECG within ±7 days | Echo-PH specific | 80,844 | 45,663 | 3,228 | 15,327 | 7.4 | 96.8 | 35.1 | 81.7 | 2.42 (2.25–2.61) | <0.001 |
| ECG within ±7 days | Echo-PH primary | 80,844 | 45,663 | 3,228 | 29,433 | 5.6 | 96.9 | 51.4 | 64.2 | 1.90 (1.77–2.04) | <0.001 |
| ECG within ±7 days | Echo RV abnormality | 80,844 | 45,663 | 3,228 | 39,537 | 5.4 | 97.4 | 66.3 | 51.8 | 2.12 (1.97–2.28) | <0.001 |

Alternative ECG–echocardiography matching windows were evaluated to assess the robustness of the main findings.

**Supplementary Table 3a. TR velocity-only diagnostic performance**

| **Outcome** | **N** | **Patients** | **Exposure positive** | **Outcome positive** | **Sensitivity** | **Specificity** | **PPV** | **NPV** | **LR+** | **LR−** |
| --- | --- | --- | --- | --- | --- | --- | --- | --- | --- | --- |
| TR velocity >2.8 m/s | 45,273 | 32,042 | 1,894 | 17,409 | 6.3 | 97.1 | 57.9 | 62.4 | 2.20 | 0.96 |
| TR velocity >3.4 m/s | 45,273 | 32,042 | 1,894 | 6,319 | 9.1 | 96.6 | 30.5 | 86.8 | 2.71 | 0.94 |

Diagnostic performance was evaluated among ECG–echocardiography pairs with available TR velocity.

**Supplementary Table 3b. TR velocity-only adjusted models**

| **Outcome** | **Model** | **OR (95% CI)** | **P value** | **N used** |
| --- | --- | --- | --- | --- |
| TR velocity >2.8 m/s | Model 1: unadjusted | 2.28 (2.08–2.50) | <0.001 | 45,273 |
| TR velocity >2.8 m/s | Model 2: age + sex + race | 2.50 (2.26–2.76) | <0.001 | 45,272 |
| TR velocity >2.8 m/s | Model 3: demographics + comorbidities + ICU | 1.89 (1.70–2.09) | <0.001 | 45,272 |
| TR velocity >3.4 m/s | Model 1: unadjusted | 2.88 (2.60–3.19) | <0.001 | 45,273 |
| TR velocity >3.4 m/s | Model 2: age + sex + race | 3.11 (2.79–3.45) | <0.001 | 45,272 |
| TR velocity >3.4 m/s | Model 3: demographics + comorbidities + ICU | 2.39 (2.14–2.67) | <0.001 | 45,272 |

Adjusted models used the same covariate structure as the main analysis.

**Supplementary Table 4. Adjusted marginal probabilities, risk differences, and risk ratios**

| **Outcome** | **Adjusted probability without RAD/RVH, % (95% CI)** | **Adjusted probability with RAD/RVH, % (95% CI)** | **Adjusted risk difference, percentage points (95% CI)** | **Adjusted risk ratio (95% CI)** | **ECG–echo pairs** | **Patients** | **Events** | **Bootstrap resamples** |
| --- | --- | --- | --- | --- | --- | --- | --- | --- |
| Echo-PH specific | 18.8 (18.4–19.2) | 30.0 (28.3–31.7) | 11.2 (9.5–12.9) | 1.59 (1.50–1.69) | 68,905 | 42,078 | 13,321 | 300 |
| Echo-PH primary | 36.2 (35.8–36.6) | 46.9 (45.1–48.7) | 10.7 (8.8–12.5) | 1.29 (1.24–1.35) | 68,905 | 42,078 | 25,268 | 300 |
| Echo RV abnormality | 48.2 (47.7–48.6) | 60.9 (58.9–62.8) | 12.7 (10.7–14.6) | 1.26 (1.22–1.30) | 68,905 | 42,078 | 33,525 | 300 |
| TR velocity >2.8 m/s | 24.9 (24.5–25.2) | 34.2 (32.3–36.0) | 9.3 (7.4–11.1) | 1.37 (1.30–1.45) | 68,905 | 42,078 | 17,409 | 300 |
| TR velocity >3.4 m/s | 8.8 (8.5–9.0) | 17.0 (15.4–18.5) | 8.3 (6.7–9.8) | 1.94 (1.78–2.12) | 68,905 | 42,078 | 6,319 | 300 |
| RV dysfunction | 15.5 (15.2–15.9) | 29.5 (27.7–31.2) | 14.0 (12.3–15.7) | 1.90 (1.79–2.02) | 68,905 | 42,078 | 11,178 | 300 |
| RV dilation | 13.0 (12.7–13.3) | 23.8 (22.1–25.5) | 10.8 (9.0–12.5) | 1.83 (1.70–1.97) | 68,905 | 42,078 | 9,315 | 300 |
| RA enlargement | 30.3 (30.0–30.7) | 34.5 (32.8–36.1) | 4.2 (2.4–5.8) | 1.14 (1.08–1.19) | 68,905 | 42,078 | 21,021 | 300 |
| Moderate/severe TR | 20.3 (19.9–20.6) | 31.0 (29.2–32.8) | 10.8 (9.0–12.5) | 1.53 (1.44–1.62) | 68,905 | 42,078 | 14,298 | 300 |

Adjusted marginal probabilities were estimated from fully adjusted logistic regression models. Confidence intervals were obtained using patient-level cluster bootstrap resampling with 300 resamples.

**Supplementary Table 5. Diagnostic performance of machine-reported RAD/RVH for component right-heart outcomes**

| **Outcome** | **N** | **TP** | **FP** | **FN** | **TN** | **Sensitivity, % (95% CI)** | **Specificity, % (95% CI)** | **PPV, % (95% CI)** | **NPV, % (95% CI)** | **LR+ (95% CI)** | **LR− (95% CI)** |
| --- | --- | --- | --- | --- | --- | --- | --- | --- | --- | --- | --- |
| RV dysfunction | 68,905 | 1,082 | 1,733 | 10,096 | 55,994 | 9.7 (9.1–10.2) | 97.0 (96.8–97.1) | 38.4 (36.6–40.3) | 84.7 (84.4–85.0) | 3.23 (3.00–3.48) | 0.93 (0.93–0.94) |
| RV dilation | 68,905 | 809 | 2,006 | 8,506 | 57,584 | 8.7 (8.1–9.3) | 96.6 (96.5–96.8) | 28.7 (27.1–30.4) | 87.1 (86.9–87.4) | 2.58 (2.38–2.79) | 0.94 (0.94–0.95) |
| RA enlargement | 68,905 | 1,025 | 1,790 | 19,996 | 46,094 | 4.9 (4.6–5.2) | 96.3 (96.1–96.4) | 36.4 (34.6–38.2) | 69.7 (69.4–70.1) | 1.30 (1.21–1.41) | 0.99 (0.98–0.99) |
| Moderate/severe TR | 68,905 | 1,039 | 1,776 | 13,259 | 52,831 | 7.3 (6.8–7.7) | 96.7 (96.6–96.9) | 36.9 (35.1–38.7) | 79.9 (79.6–80.2) | 2.23 (2.07–2.41) | 0.96 (0.95–0.96) |

Diagnostic performance for component right-heart outcomes was calculated at the ECG–echocardiography pair level.

**Supplementary Table 6. Validation of automated ECG text extraction**

| **Validation target** | **Reviewed reports** | **TP** | **FP** | **FN** | **TN** | **PPV, % (95% CI)** | **NPV, % (95% CI)** | **Sensitivity, % (95% CI)** | **Specificity, % (95% CI)** | **Accuracy, % (95% CI)** |
| --- | --- | --- | --- | --- | --- | --- | --- | --- | --- | --- |
| RAD/RVH composite | 200 | 91 | 9 | 0 | 100 | 91.0 (83.6–95.8) | 100.0 (96.4–100.0) | 100.0 (96.0–100.0) | 91.7 (84.9–96.2) | 95.5 (91.6–97.9) |
| RAD | 200 | 79 | 0 | 0 | 121 | 100.0 (95.4–100.0) | 100.0 (97.0–100.0) | 100.0 (95.4–100.0) | 100.0 (97.0–100.0) | 100.0 (98.2–100.0) |
| RVH | 200 | 22 | 12 | 0 | 166 | 64.7 (46.5–80.3) | 100.0 (97.8–100.0) | 100.0 (84.6–100.0) | 93.3 (88.5–96.5) | 94.0 (89.8–96.9) |

A random sample of 200 ECG report texts, including 100 RAD/RVH-positive and 100 RAD/RVH-negative reports according to the automated extraction, was independently reviewed using predefined adjudication rules. Definite mentions of right axis deviation, rightward axis, right ventricular hypertrophy, or RVH were classified as positive. Borderline, possible, probable, or otherwise uncertain statements were not considered definite positive findings.

**Supplementary Table 7. Discordant cases in ECG text extraction validation**

| **Validation ID** | **Automated RAD** | **Automated RVH** | **Automated RAD/RVH** | **Reviewed RAD** | **Reviewed RVH** | **Reviewed RAD/RVH** | **Uncertain/borderline only** | **Negated only** | **Comment** |
| --- | --- | --- | --- | --- | --- | --- | --- | --- | --- |
| VAL002 | 0 | 1 | 1 | 0 | 0 | 0 | 1 | 0 | Uncertain/probable RVH only |
| VAL026 | 0 | 1 | 1 | 0 | 0 | 0 | 1 | 0 | Uncertain/probable RVH only |
| VAL036 | 0 | 1 | 1 | 0 | 0 | 0 | 1 | 0 | Uncertain/probable RVH only |
| VAL040 | 0 | 1 | 1 | 0 | 0 | 0 | 1 | 0 | Uncertain/probable RVH only |
| VAL057 | 0 | 1 | 1 | 0 | 0 | 0 | 1 | 0 | Uncertain/probable RVH only |
| VAL063 | 0 | 1 | 1 | 0 | 0 | 0 | 1 | 0 | Uncertain/probable RVH only |
| VAL068 | 0 | 1 | 1 | 0 | 0 | 0 | 1 | 0 | Uncertain/probable RVH only |
| VAL073 | 0 | 1 | 1 | 0 | 0 | 0 | 1 | 0 | Uncertain/probable RVH only |
| VAL085 | 0 | 1 | 1 | 0 | 0 | 0 | 1 | 0 | Uncertain/probable RVH only |

Discordant cases were defined as reports for which the automated RAD/RVH classification differed from the independent review. All discordant cases were automated RVH-positive reports containing uncertain RVH wording.

**Supplementary Table 8. ECG–echocardiography interval and TR velocity availability**

| **Item** | **Value** |
| --- | --- |
| ECG–echocardiography pairs | 68,905 |
| Patients | 42,078 |
| RAD/RVH-positive pairs | 2,815 |
| TR velocity available | 45,273 (65.7%) |
| TR velocity missing | 23,632 (34.3%) |
| Median absolute ECG–echocardiography interval, hours | 19.7 |
| Interquartile range of absolute interval, hours | 5.6–45.1 |
| Same-day ECG–echocardiography pairs | 40,036 (58.1%) |
| ECG–echocardiography pairs within 6 hours | 17,980 (26.1%) |

The primary analysis used the closest ECG obtained from 7 days before echocardiography through the time of echocardiography. TR velocity availability was calculated at the ECG–echocardiography pair level.

**Supplementary Figure Legends**

**Supplementary Figure 1. Diagnostic performance of ECG findings for selected echocardiographic outcomes.**

Diagnostic performance of machine-reported ECG findings is shown for Echo-PH specific and Echo RV abnormality. Performance metrics include sensitivity, specificity, positive predictive value, and negative predictive value.


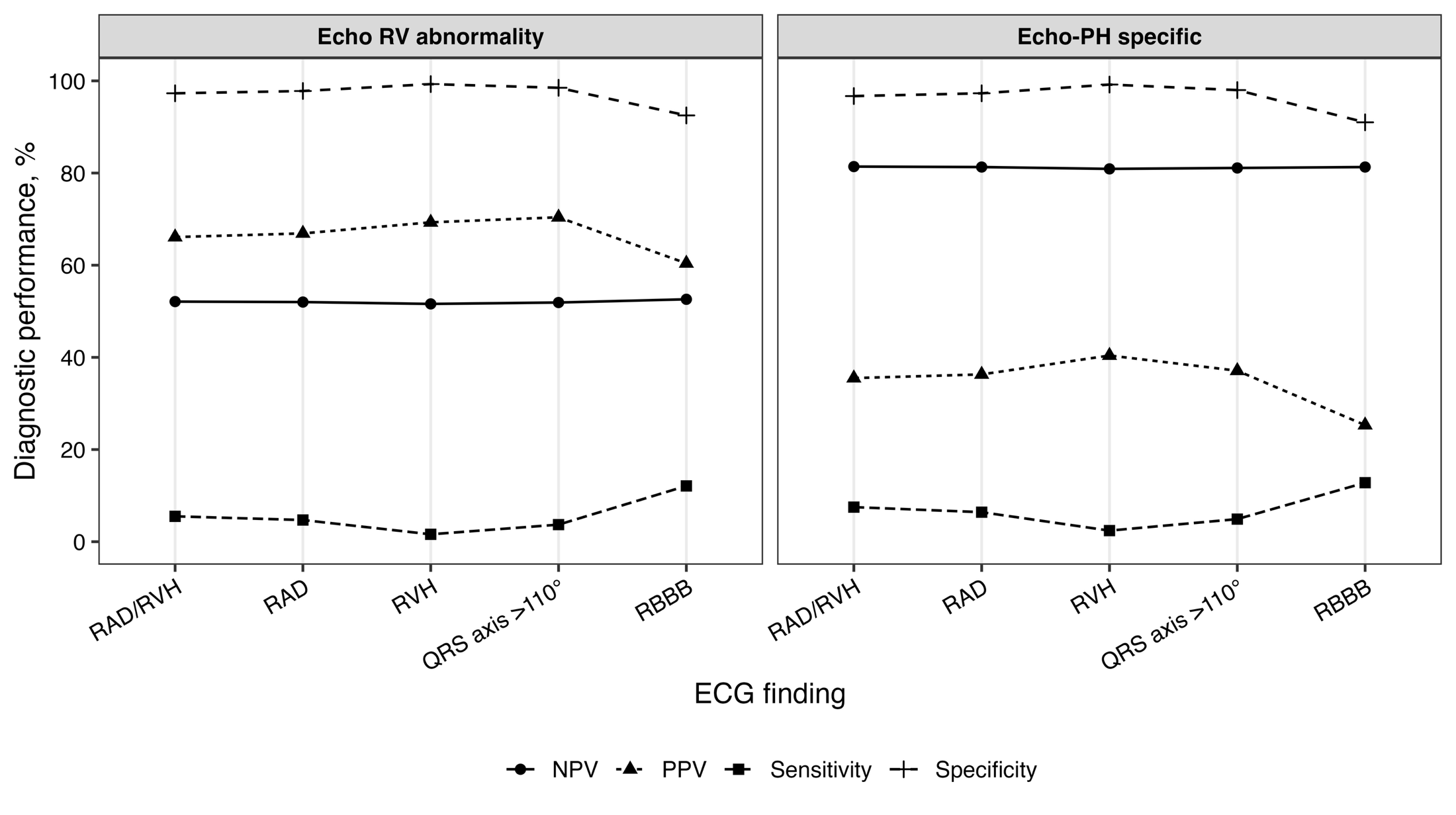


**Supplementary Figure 2. Time-window sensitivity analysis.**

Unadjusted odds ratios and 95% confidence intervals are shown for machine-reported RAD/RVH across alternative ECG–echocardiography matching windows for selected echocardiographic outcomes.


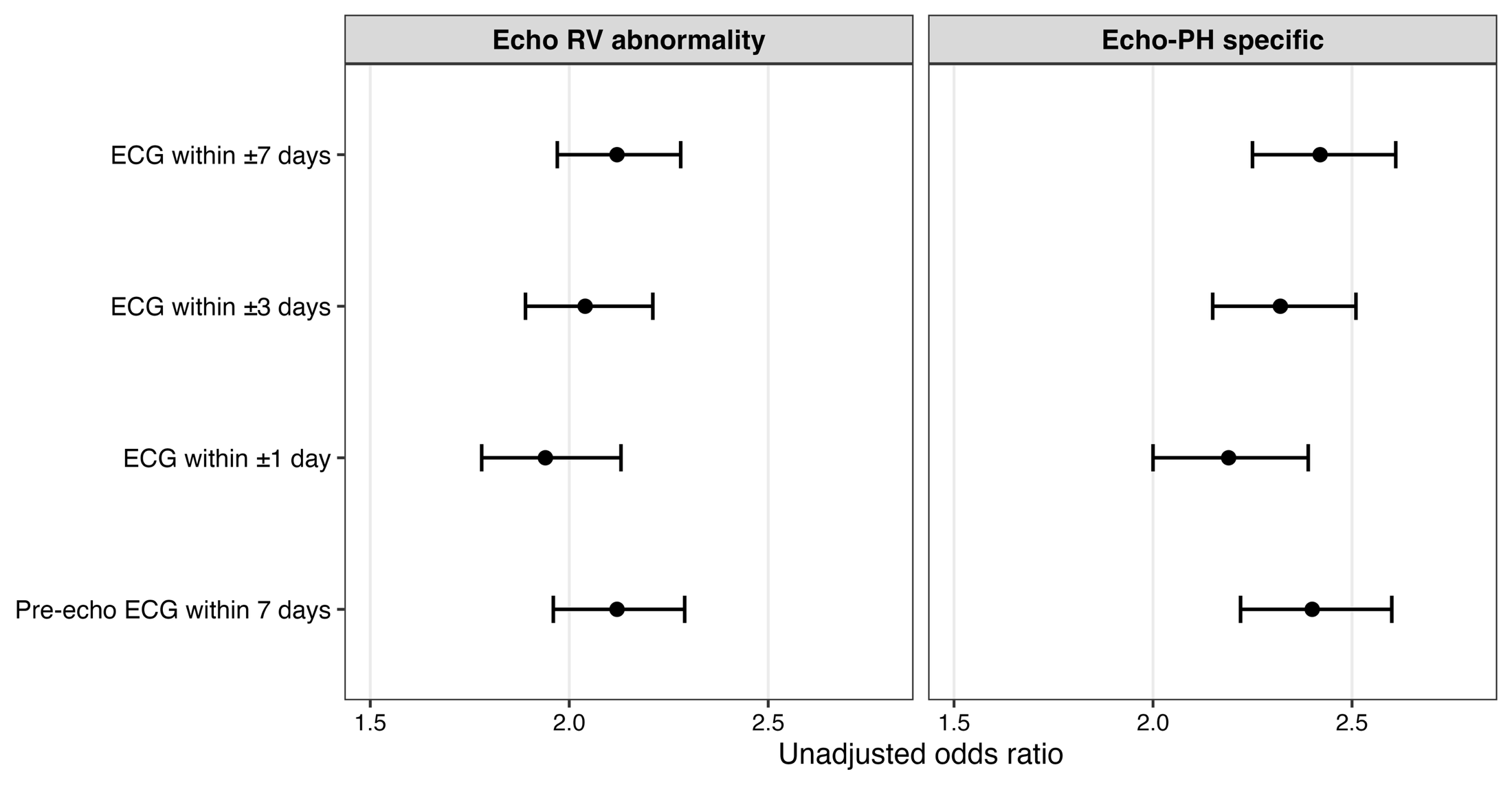


**Supplementary Figure 3. Patient-level and TR velocity-only sensitivity analyses.**

Adjusted odds ratios and 95% confidence intervals are shown for patient-level sensitivity analyses and TR velocity-only sensitivity analyses.


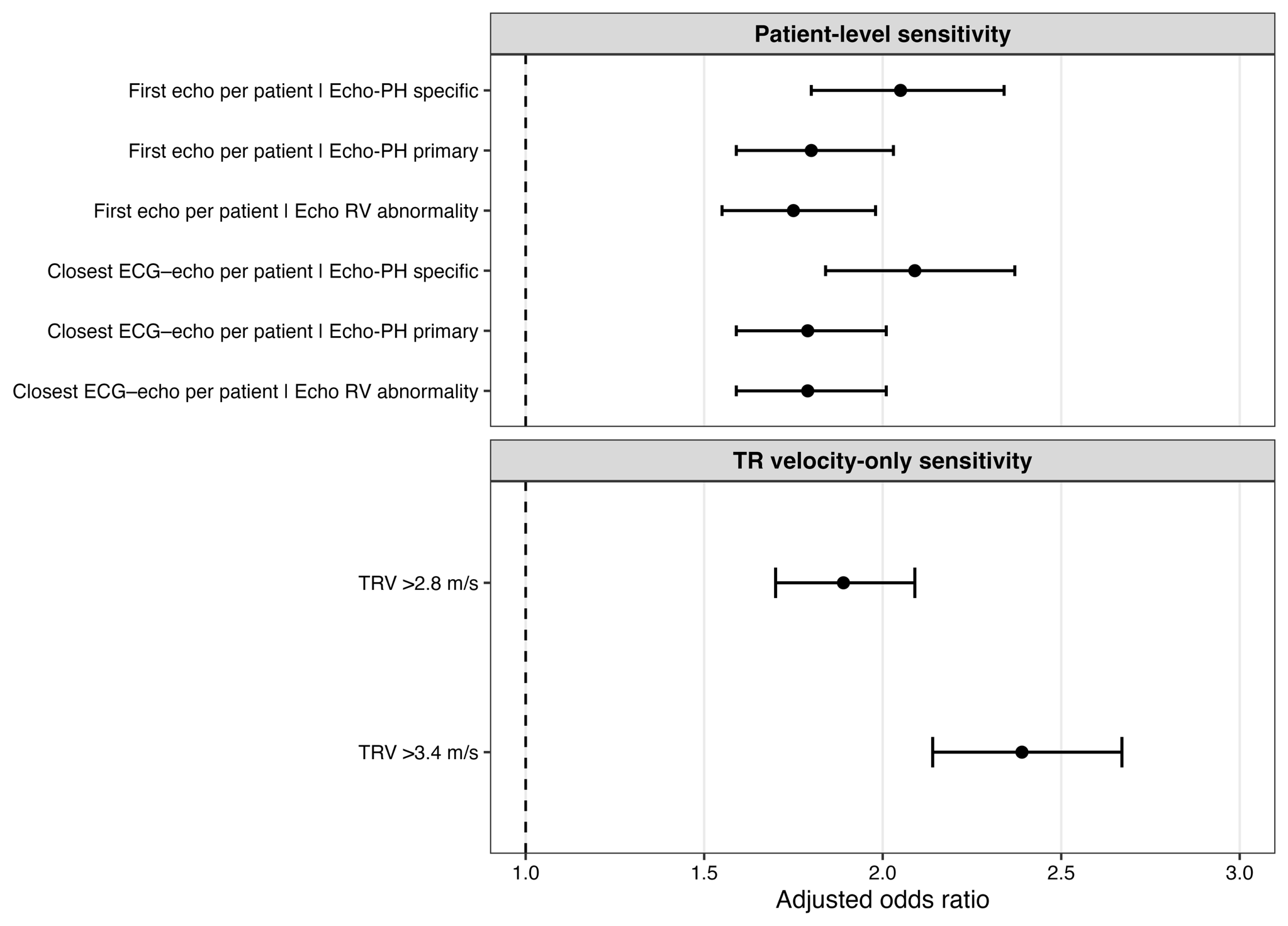

Supplement: Supplementary file 1 — Supplementary Table 1: Patient‐level sensitivity analysis. Supplementary Table 2: Time‐window sensitivity analysis. Supplementary Table 3a TR velocity‐only diagnostic performance. Supplementary Table 3b: TR velocity‐only adjusted models. Supplementary Table 4: Adjusted marginal probabilities, risk differences, and risk ratios. Supplementary Table 5: Diagnostic performance of machine‐reported RAD/RVH for component right‐heart outcomes. Supplementary Table 6: Validation of automated ECG text extraction. Supplementary Table 7: Discordant cases in ECG text extraction validation. Supplementary Table 8: ECGechocardiography interval and TR velocity availability. Supplementary Figure 1: Diagnostic performance of ECG findings for selected echocardiographic outcomes. Diagnostic performance of machine‐reported ECG findings is shown for Echo‐PH specific and Echo RV abnormality. Performance metrics include sensitivity, specificity, positive predictive value, and negative predictive value. Supplementary Figure 2: Time‐window sensitivity analysis. Unadjusted odds ratios and 95% confidence intervals are shown for machine‐reported RAD/RVH across alternative ECGechocardiography matching windows for selected echocardiographic outcomes. Supplementary Figure 3: Patient‐level and TR velocity‐only sensitivity analyses. Adjusted odds ratios and 95% confidence intervals are shown for patient‐level sensitivity analyses and TR velocity‐only sensitivity analyses. [file ECHO-43-e70539-s001.docx]
